# Construction of prediction models for novel subtypes in patients with arteriosclerosis obliterans undergoing endovascular therapy: an unsupervised machine learning study

**DOI:** 10.1186/s13019-024-02913-6

**Published:** 2024-06-25

**Authors:** Xiaocheng Li, Lin Zhang, Que Li, Jiangfeng Zhang, Xiao Qin

**Affiliations:** Department of Vascular Surgery Ward, The First Affiliated Hospital of Guangxi Medical University, No.6 of Shuangyong Road, Nanning, Guangxi 530021 P. R. China

**Keywords:** Machine learning, Arteriosclerosis obliterans, Endovascular therapy, Cluster analysis, Prediction model

## Abstract

**Background:**

Arteriosclerosis obliterans (ASO) is a chronic arterial disease that can lead to critical limb ischemia. Endovascular therapy is increasingly used for limb salvage in ASO patients, but the outcomes vary. The development of prediction models using unsupervised machine learning may lead to the identification of novel subtypes to guide patient prognosis and treatment.

**Methods:**

This retrospective study analyzed clinical data from 448 patients with ASOs who underwent endovascular therapy. Unsupervised machine learning algorithms were employed to identify subgroups. To validate the precision of the clustering outcomes, an analysis of the postoperative results of the clusters was conducted. A prediction model was constructed using binary logistic regression.

**Results:**

Two distinct subgroups were identified by unsupervised machine learning and characterized by differing patterns of clinical features. Patients in Cluster 2 had significantly worse conditions and prognoses than those in Cluster 1. For the novel ASO subtypes, a nomogram was developed using six predictive factors, namely, platelet count, ankle brachial index, Rutherford category, operation method, hypertension, and diabetes status. The nomogram achieved excellent discrimination for predicting membership in the two identified clusters, with an area under the curve of 0.96 and 0.95 in training cohort and internal test cohort.

**Conclusion:**

This study demonstrated that unsupervised machine learning can reveal novel phenotypic subgroups of patients with varying prognostic risk who underwent endovascular therapy. The prediction model developed could support clinical decision-making and risk counseling for this complex patient population. Further external validation is warranted to assess the generalizability of the findings.

**Supplementary Information:**

The online version contains supplementary material available at 10.1186/s13019-024-02913-6.

## Background

Peripheral Arterial Disease (PAD) is a prevalent circulatory condition marked by narrowed arteries, which impede blood flow to the limbs [1]. Arteriosclerosis Obliterans (ASO) constitutes a significant subset of PAD, representing the chronic advancement of arteriosclerotic disease. Patients with ASO typically present with symptoms ranging from intermittent claudication to more severe manifestations such as rest pain and tissue loss, reflecting varying stages of disease severity [2]. Endovascular therapy has emerged as a promising approach for treating ASO patients, offering a less invasive alternative to traditional surgical procedures. However, significant variability exists in patient responses to this therapy, and accurately predicting outcomes remains challenging [3].

In recent years, advances in unsupervised machine learning (UMLA) techniques have opened new avenues for understanding the heterogeneity within ASO patients. These techniques facilitate the automatic clustering of patients into distinct subtypes based on diverse clinical and omics data [4]. This study aimed to evaluate the power of the UMLA to construct a prediction model that can identify novel subtypes within the ASO patient population undergoing endovascular therapy.

In this study, clinical data from ASO patients who underwent endovascular therapy were collected. Using UMLA, patient clustering into distinct groups based on their clinical characteristics was performed. Subsequently, a comparative analysis of surgical outcomes and postoperative complications between the identified patient groups was carried out to validate the accuracy of clustering. Finally, we examined the potential risk factors contributing to patient clustering with the aim of constructing a novel prediction model for ASO subtypes. This prediction model has the potential to guide clinicians in identifying patients at risk for poor outcomes, facilitating timely intervention and tailored therapeutic approaches.

## Methods

### Patients

This study is a single-center retrospective study. Clinical data were collected from patients diagnosed with ASO who underwent endovascular treatment for lower extremity conditions at Guangxi Medical University between January 2015 and January 2023. The inclusion criteria for patients were as follows: had (a) met the established diagnostic criteria for ASO in the lower limbs [5], (b) had undergone endovascular treatment for the lower extremities, (c) received ultrasound, computed tomography angiography (CTA), or digital subtraction angiography (DSA) to assess lower extremity artery occlusion, and (d) had complete clinical data available. The exclusion criteria included (a) loss to follow-up and (b) the presence of a malignant tumor. Based on the inclusion and exclusion criteria, a total of 448 eligible patients were included in the study.

We collected 18 preoperative variables and 10 postoperative variables. The 18 preoperative variables included sex, age, body mass index (BMI), total cholesterol (TC), triglyceride (TG), low-density lipoprotein cholesterol (LDL-C), platelet count, ankle brachial index (ABI) [6], Rutherford category [7], TransAtlantic Inter-Society Consensus (TASC) II classification [8], operative method, operation time and history of smoking, hypertension, diabetes, cardiovascular heart disease (CAD), cerebrovascular disease (CVD) and chronic kidney disease (CKD). The 10 postoperative variables were the degree of claudication and tissue loss, ischemic rest pain, multiple organ dysfunction syndrome (MODS), renal failure, wound infection, wound ulceration, amputation, death in hospital, and septicemia. All the postoperative outcomes were assessed during the 30-day follow-up.

This study was conducted in accordance with the Declaration of Helsinki and was approved by the Ethics Committee of The First Affiliated Hospital of Guangxi Medical University.

### Clustering clinical data using UMLA

**Fig. 1:**
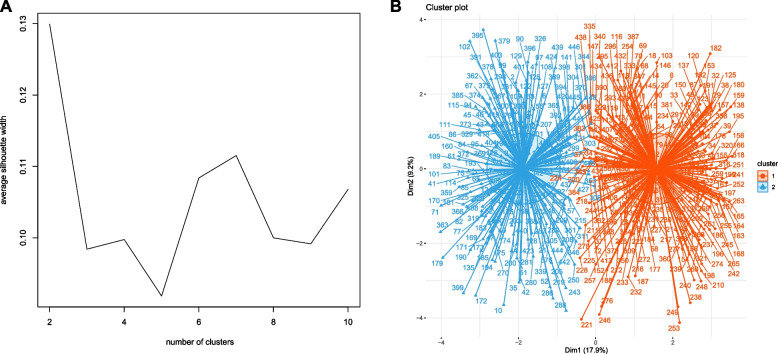
Result of unsupervised machine learning. **A** Optimal clustering number of the K-means clustering algorithm was determined by Silhouette coefficient (SC). The peak of the curve is the best value for the Silhouette coefficient (Y-axis); the best number of clusters was equal to 2 (X–axis). **B** Scatter plots of patients’ clinical data. Scatter points on the graph represent each patient. The K-means algorithm divides patients into two clusters. The red scatter represents cluster 1 and the blue scatter represents cluster 2

K-means clustering algorithm was used to cluster the ASO patients. K-means clustering is a widely used unsupervised learning method for discovering distinct groupings in data by minimizing within-cluster variation. The algorithm steps are as follows: (a) randomly initialize k cluster centroids; (b) assign each data point to the nearest cluster; (c) recompute cluster centroids based on assigned points; and (d) repeat steps 2–3 until the centroids no longer change or the maximum number of iterations is reached [9]. Figure 1A shows the K-means algorithm flow. R software version 4.2.1 employed the scale function from the ‘factoextra’ package to standardize the preoperative variables of ASO patients [10]. The optimal number of clusters (K-value) was determined using the ‘fpc’ package, which calculated the silhouette coefficient (SC). The silhouette coefficient is a metric utilized to assess the clustering performance of an unsupervised learning model, measuring the degree to which each data point fits into its assigned cluster [11].

$$\text{SC}\left(i\right)=\frac{b\left(i\right)-a\left(i\right)}{max\left\{a\left(i\right),b\left(i\right)\right\}}$$

Where SC $\left(i\right)$ denotes a calculated score that evaluates the quality of the cluster. For each data point $i$, a $\left(i\right)$ constitutes the average distance from i to all other points within its cluster, while b $\left(i\right)$ represents the minimum average distance $i$ of to points in any different cluster. The overall SC of the model is determined by averaging the silhouette scores of all data points. Scores approaching + 1 suggest dense, well-separated clusters; scores around 0 imply overlapping clusters; and negative scores may signify incorrect assignment of points to clusters. In essence, SC serves as a quantitative measure for assessing the suitability of data clustering by a specific model and is instrumental in evaluating and refining the configuration of unsupervised learning clustering algorithms [12].

Based on the preoperative data, patients were divided into two clusters according to the UMLA, after which the differences in the postoperative data between the two clusters were analyzed to verify the accuracy of the UMLA clustering.

### Construction of the prediction model

The dataset was randomly divided into training and validation cohorts at a ratio of 7:3, and the variables were compared. In the training cohort, the least absolute shrinkage and selection operator (LASSO) logistic regression analysis was used for multivariate analysis to screen the independent risk factors. The training dataset was then utilized to develop a multivariate logistic regression model. The coefficients from this model were employed to construct the nomogram. The nomogram mapped the logistic regression coefficients to a 0–100 scale to provide a visual representation of the predicted probabilities [13]. The performance of the nomogram was assessed using the receiver operating characteristic (ROC) curve and calibration curve, with the area under the ROC curve (AUC) ranging from 0.5 (no discriminant) to 1 (complete discriminant). Additionally, a decision curve analysis (DCA) was conducted to establish the net benefit threshold for prediction.

### Statistical analysis

IBM SPSS 26.0 and R 4.2.1 software were used for statistical analysis. Clinical data are presented as the mean (SD) and median (P25, P75). Depending on the data type, Student’s t-test, the Mann–Whitney U test, or the chi-square test was performed. A *p*-value < 0.05 was considered to indicate statistical significance.

## Results

### Results of UMLA

**Fig. 2:**
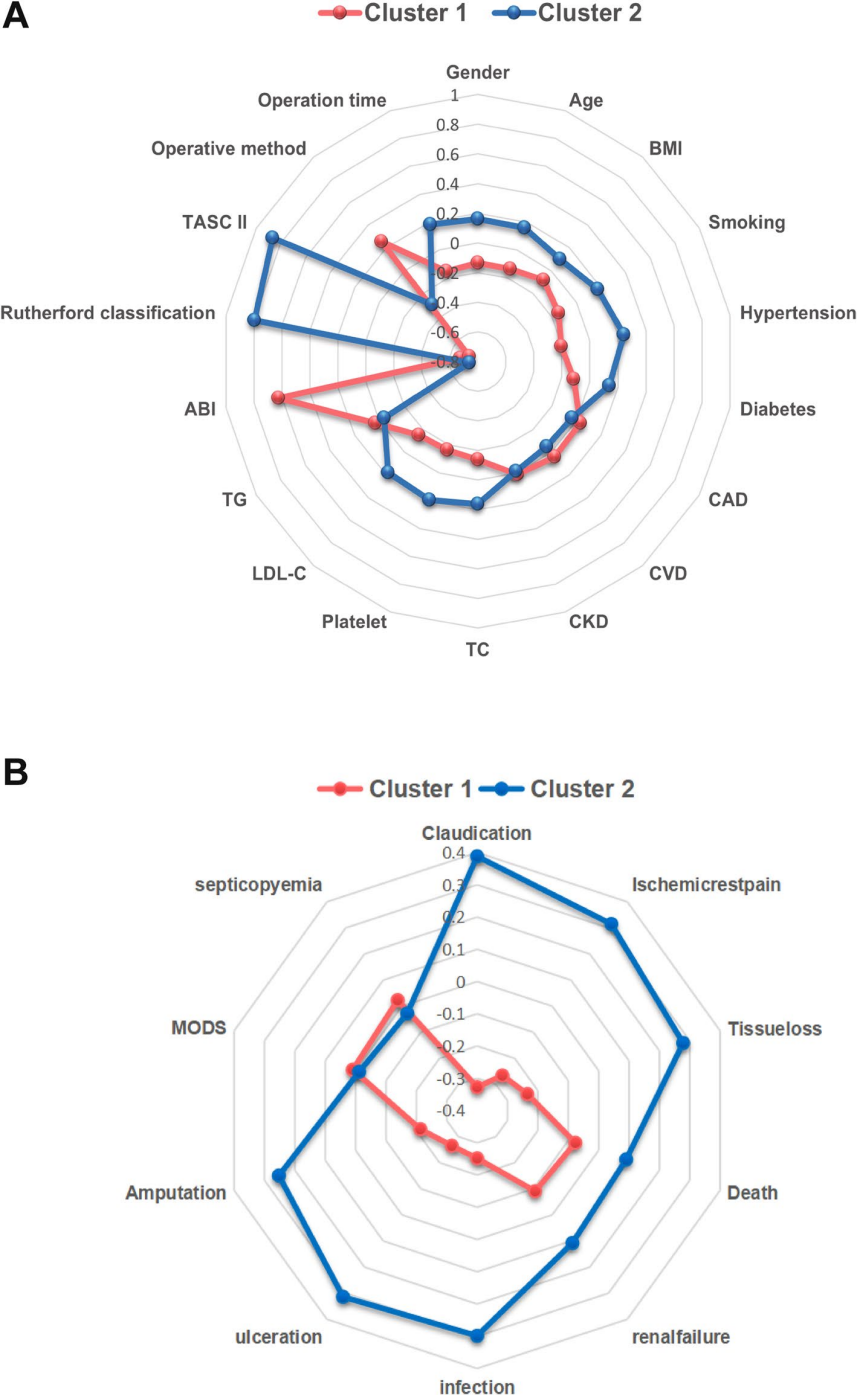
Radargram of LE-PAD patients’ preoperative and postoperative variables in two clusters. **A** Radargram of LE-PAD patients’ preoperative variables in two clusters. **B** Radargram of LE-PAD patients’ postoperative variables in two clusters. ABI: Ankle brachial index; BMI: Body mass index; CAD: Coronary artery disease; CVD:Cerebrovascular disease; CKD: Chronic kidney disease; LDL-C: Low-density lipoprotein cholesterol; MODS: multiple organ dysfunction syndrome; PTA: Percutaneous transluminal angioplasty; TASC II: Inter-society consensus for the management of peripheral arterial disease; TC: Total cholesterol; TG: triglyceride

Figure 1B displays the optimal clustering number determined by the K-means algorithm, with the peak of the curve indicating the best value for the SC (Y-axis) [14]. This suggests that two is the optimal number of clusters. Consequently, the algorithm effectively clustered the current clinical data into two clusters (Fig. 1C). Table 1 presents the K-means clustering results for the clinical data. The sex distribution was significantly different between the two clusters (*p* = 0.002), with cluster 2 having a greater proportion of males (71%) than cluster 1 (56%). Age, body mass index (BMI), total cholesterol (TC), platelet count, low-density lipoprotein cholesterol (LDL-C), Rutherford category, smoking status, hypertension, and diabetes incidence were significantly greater in cluster 2 than in cluster 1 (*p* < 0.05). The ankle brachial index (ABI) in cluster 2 was significantly lower than that in cluster 1 (*p* < 0.001). According to the TransAtlantic Inter-Society Consensus (TASC) II classification, a greater proportion of patients with type C and D lesions was observed in cluster 2, while type A and B lesions were more prevalent in cluster 1 (*p* < 0.001). Furthermore, significant differences existed between the two clusters in terms of the operation method and duration. No significant differences were noted in the other preoperative variables. Figure 2A displays the radargram of the preoperative variables.

**Table 1:** Preoperative conditions of the study patients by clusters

Characteristic	cluster		*p*-value
	1, *N* = 243	2, *N* = 205	
**Age**			0.001
Mean ± SD	58 ± 12	61 ± 12	
Median (IQR)	56 (49, 68)	62 (52, 70)	
**BMI**			0.007
Mean ± SD	22.5 ± 3.8	23.1 ± 3.3	
Median (IQR)	22.0 (20.0, 24.6)	23.1 (20.5, 25.5)	
**TC**			0.002
Mean ± SD	7.5 ± 3.6	8.7 ± 4.3	
Median (IQR)	6.7 (5.1, 9.1)	7.5 (5.9, 10.3)	
**Platelet**			0.009
Mean ± SD	210 ± 79	247 ± 123	
Median (IQR)	209 (154, 257)	227 (165, 285)	
**LDL-C**			0.009
Mean ± SD	5.40 ± 2.67	6.48 ± 3.77	
Median (IQR)	4.74 (3.60, 7.17)	5.79 (3.62, 8.48)	
**TG**			0.618
Mean ± SD	1.55 ± 0.66	1.50 ± 0.62	
Median (IQR)	1.56 (1.06, 1.89)	1.51 (1.06, 1.89)	
**ABI**			< 0.001
Mean ± SD	0.64 ± 0.13	0.39 ± 0.15	
Median (IQR)	0.67 (0.55, 0.74)	0.38 (0.29, 0.46)	
**Rutherford category**			< 0.001
Mean ± SD	2.35 ± 0.76	4.58 ± 1.29	
Median (IQR)	2.00 (2.00, 2.00)	5.00 (4.00, 6.00)	
**Gender**			0.002
Female	106 (44%)	60 (29%)	
Male	137 (56%)	145 (71%)	
**Smoking**			< 0.001
No	102 (42%)	55 (27%)	
Yes	141 (58%)	150 (73%)	
**Hypertension**			< 0.001
No	178 (73%)	106 (52%)	
Yes	65 (27%)	99 (48%)	
**Diabetes**			0.007
No	196 (81%)	143 (70%)	
Yes	47 (19%)	62 (30%)	
**CAD**			0.478
No	226 (93%)	194 (95%)	
Yes	17 (7%)	11 (5%)	
**CVD**			0.348
No	226 (93%)	195 (95%)	
Yes	17 (7%)	10 (5%)	
**CKD**			0.802
No	220 (91%)	187 (91%)	
Yes	23 (9%)	18 (9%)	
**TASC II**			< 0.001
A	121 (50%)	4 (2%)	
B	107 (44%)	14 (7%)	
C	15 (6%)	117 (57%)	
D	0 (0%)	70 (34%)	
**Operative method**			< 0.001
PTA	38 (16%)	109 (53%)	
Atherectomy + PTA	124 (51%)	41 (20%)	
Atherectomy + PTA + Stent	81 (33%)	55 (27%)	
**Operation time**			< 0.001
< 2h	76 (31%)	52 (25%)	
2 ~ 4h	124 (51%)	78 (38%)	
> 4h	43 (18%)	75 (37%)	

*ABI* Ankle brachial index, *BMI* Body mass index, *CAD* Coronary artery disease, *CVD* Cerebrovascular disease, *CKD* Chronic kidney disease, *LDL-C* Low-density lipoprotein cholesterol, *PTA* Percutaneous transluminal angioplasty, *TASC II* TransAtlantic Inter-Society Consensus II, *TC* Total cholesterol, *TG* triglyceride

### Comparison of postoperative variables between the two clusters

Table 2 illustrates the differences in postoperative variables between the two clusters. Cluster 2 exhibited a greater incidence of renal failure than did cluster 2 (*p* = 0.036). Furthermore, the incidence of wound infection, ulceration, and amputation in cluster 2 was significantly greater than that in cluster 1 (*p* < 0.001). Additionally, claudication, ischemic rest pain, and tissue loss were more severe in cluster 2 than in cluster 1 (*p* < 0.001). These results indicated worse prognoses and outcomes for patients in cluster 2 than for those in cluster 1 (Fig. 2B). The differences in postoperative variables between the two clusters confirmed the accuracy of UMLA clustering in this study. Hence, UMLA successfully stratified ASO patients into severe and mild cohorts based on the acquired clinical data.

**Table 2:** Postoperative conditions of patients in two clusters

Characteristic	cluster		*p*-value
	1, *N* = 243	2, *N* = 205	
**Claudication**			< 0.001
Asymptomatic	222 (91%)	114 (56%)	
Mild	12 (5%)	55 (27%)	
Moderate	6 (2%)	26 (13%)	
Severe	3 (1%)	10 (5%)	
**Ischemic rest pain**			< 0.001
No	234 (96%)	158 (77%)	
Yes	9 (4%)	47 (23%)	
**Tissue loss**			< 0.001
No	231 (95%)	152 (74%)	
Minor	7 (3%)	43 (21%)	
Major	5 (2%)	10 (5%)	
**MODS**			0.825
No	235 (97%)	199 (97%)	
Yes	8 (3%)	6 (3%)	
**Renal failure**			0.036
No	238 (98%)	193 (94%)	
Yes	5 (2%)	12 (6%)	
**Infection**			< 0.001
No	237 (98%)	166 (81%)	
Yes	6 (2%)	39 (19%)	
**Ulceration**			< 0.001
No	241 (99%)	171 (83%)	
Yes	2 (1%)	34 (17%)	
**Amputation**			< 0.001
No	226 (93%)	157 (77%)	
Yes	17 (7%)	48 (23%)	
**Death**			0.077
No	236 (97%)	192 (94%)	
Yes	7 (3%)	13 (6%)	
**Septicopyemia**			0.592
No	235 (97%)	200 (98%)	
Yes	8 (3%)	5 (2%)	

*MODS* multiple organ dysfunction syndrome

### Construction of a prediction model for the novel ASO subtype

**Fig. 3:**
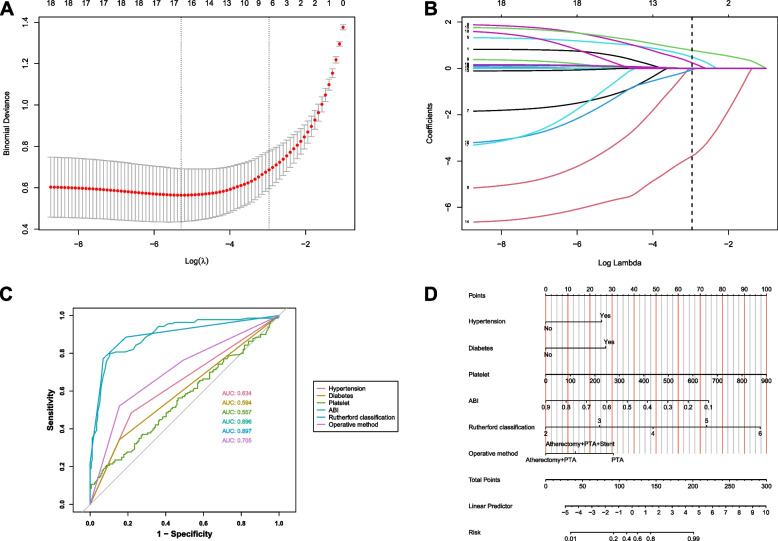
Construction of the predictive model. **A** Cross-validation plot of the LASSO regression model. **B** Coefficient path plot of the LASSO regression model. **C** ROC curve analysis of the six candidate diagnostic indicators. **D** Nomogram for prediction model. ABI: Ankle brachial index; AUC: Area under the curve; PTA: Percutaneous transluminal angioplasty

**Fig. 4:**
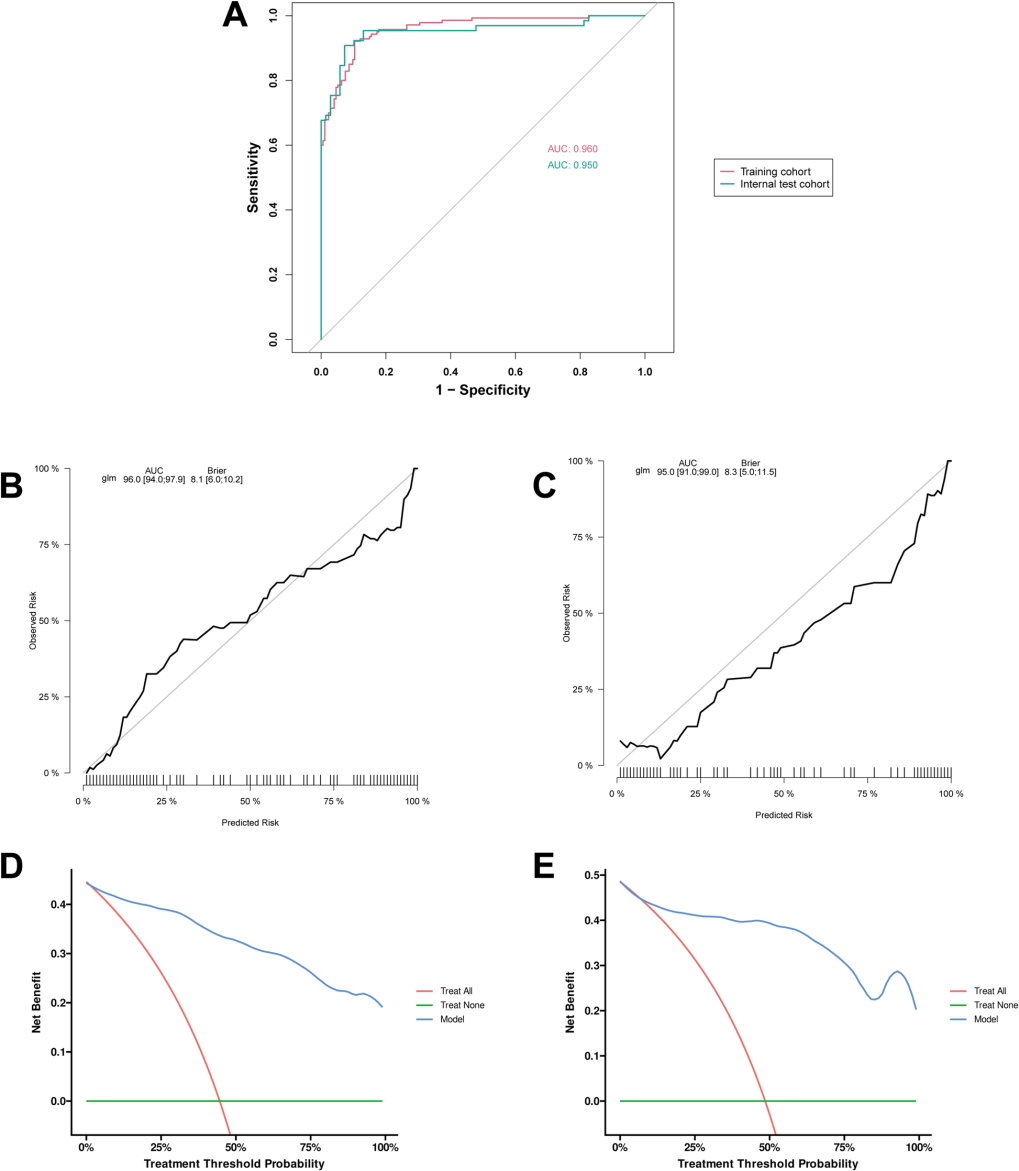
Validation of the predictive model of the predictive model. **A** ROC curves of the nomogram predictive model. **B** Calibration curve of the nomogram predictive mode for the training cohort. **C** Calibration curve of the nomogram prediction mode for the internal test cohort. **D** Decision curve analysis of the nomogram of the training cohort. **E** Decision curve analysis of the nomogram of the internal test cohort. AUC: Area under the curve

This study examined the clinical data of 448 ASOs, divided into a Training Cohort comprising 314 individuals and an Internal Test Cohort with 134 participants. Supplementary Table 1 displays the baseline demographic and clinical features of the respective cohorts. Next, the candidate predictors were included in the original model, which were then reduced to 6 potential predictors using LASSO regression analysis performed in the training cohort. The cross-validation plot of the LASSO regression model is plotted in the Fig. 3A. A coefficient path plot is also shown in the Fig. 3B. The most regularized and parsimonious model, with a cross-validated error within one standard error of the minimum, included six variables. As shown in Fig. 3C, the ROC analysis of the abovementioned variables yielded AUC values greater than 0.5. Further multivariate logistic analyses were carried out in different clusters. Results are shown in the Table 3. The final logistic model included 6 independent predictors (hypertension, diabetes, platelet, ABI, rutherford classification, and operation method) and was developed as a simple-to-use nomogram, which is illustrated in the Fig. 3D. The AUCs of the model in the different cohorts were 0.96 and 0.95 (Fig. 4A). The calibration plots of the nomogram in the different cohorts are plotted in the Fig. 4 B-C, which demonstrate a good correlation between the observed and predicted cluster. The results showed that the original nomogram was still valid for use in the validation sets, and the calibration curve of this model was relatively close to the ideal curve, which indicates that the predicted results were consistent with the actual findings. Figure 4D-E display the DCA curves related to the nomogram. A high-risk threshold probability indicates the chance of significant discrepancies in the model’s prediction when clinicians encounter major flaws while utilizing the nomogram for diagnostic and decision-making purposes. This research shows that the nomogram offers substantial net benefits for clinical application through its DCA curve.

**Table 3:** Results of Multivariate Logistic regression for Training Cohort

Characteristic	*N*	Event N	OR^1^	95% CI^1^	*p*-value
**Hypertension**					
No	208	72	—	—	
Yes	106	68	4.02	1.68, 9.61	0.002
**Diabetes**					
No	239	92	—	—	
Yes	75	48	4.42	1.56, 12.50	0.005
**Platelet**	314	140	1.01	1.00, 1.01	0.010
**ABI**	314	140	0.01	0.00, 0.15	0.002
**Rutherford classification**	314	140	3.80	2.43, 5.94	< 0.001
**Operative method**					
PTA	100	73	—	—	
Atherectomy + PTA	121	33	0.19	0.07, 0.52	0.001
Atherectomy + PTA + Stent	93	34	0.39	0.12, 1.24	0.109

*ABI* Ankle brachial index, *PTA* Percutaneous transluminal angioplasty

## Discussion

### Clinical significance of UMLA modeling of the novel ASO subtype

With the advancement of artificial intelligence (AI), UMLA has found extensive application in clinical research. For instance, Eshaghi et al. employed UMLA to categorize multiple sclerosis patients into pathology-based subtypes utilizing magnetic resonance imaging [15]. Kung et al. identified depression subtypes using UMLA on symptom data from more than 18,000 patients [16]. These studies demonstrated the potential of using UMLA to discover novel subtypes and construct prediction models. Establishing accurate subtyping and risk prediction enables precision medicine approaches for these complex, heterogeneous diseases.

Compared to supervised machine learning and traditional predictive modeling approaches, UMLA excels in uncovering hidden patterns and structures within the data without prior labeling [17]. This is particularly advantageous in medical research where the complexity and variability of clinical data often mask underlying patterns that could be crucial for patient stratification and outcome prediction. By leveraging UMLA, we identified distinct subtypes of ASO patients that might not have been evident through traditional methods.

Additionally, unlike supervised machine learning, UMLA groups patients based on inherent similarities in their clinical features. This data-driven approach enables the identification of patient subgroups that share common characteristics and potentially similar prognostic trajectories [18]. Such stratification is essential for personalized medicine, allowing for tailored treatment strategies that could improve patient outcomes.

Furthermore, UMLA models are highly adaptable and can be applied to various types of data, including continuous, categorical, and binary variables [19]. This flexibility is beneficial in the medical field where patient data is often heterogeneous. UMLA can be continuously refined and updated as new data becomes available, ensuring that the predictive models remain relevant and accurate over time.

In the current study, UMLA categorized 18 predictor variables derived from the information of ASO patients into two distinct clusters: cluster 1 and cluster 2. Further analysis of postoperative variables in these two clusters revealed significantly worse conditions and prognoses for patients in the cluster 2 cohort. This subtyping system provides preliminary evidence that ASOs may represent multiple biological entities with varying prognostic risks.

The nomogram created in this study may have clinical utility for individual risk stratification. By incorporating a set of clinical variables, the nomogram demonstrated outstanding discrimination in predicting membership in the two identified clusters, achieving an AUCs of 0.96 and 0.95 in training cohort and internal test cohort. If validated, this model could be utilized to customize treatment strategies based on the anticipated disease trajectory. Patients at high risk might benefit from more intensive follow-up and monitoring, whereas low-risk patients could potentially avoid unnecessary interventions.

Several limitations should be noted. However, our model requires external validation in diverse patient cohorts before clinical implementation. Additionally, The k-means clustering results reveal a relatively low silhouette coefficient, albeit still within an acceptable range. This finding suggests that the severity of ASO among patients likely exists along a spectrum rather than discrete clusters. Future studies could benefit from incorporating additional variables, including genetic, biomarker, and imaging data, to enhance clustering performance [20]. Nonetheless, this study offers a proof-of-concept for an AI-driven approach to unraveling heterogeneity and facilitating personalized medicine in ASO patients. With additional validation and fine-tuning, machine learning models could be integrated into clinical decision support systems to assist in tailoring individualized treatments. In summary, advanced analytics hold significant potential for discovering new insights into the disease and enhancing outcomes for this intricate vascular disorder.

### Analysis of risk factors for poor prognosis after endovascular therapy in patients with ASO

In this study, hypertension, diabetes status, high platelet count, low ABI and advanced Rutherford category were identified as independent risk factors for poor prognosis after endovascular therapy in patients who underwent ASO. On the contrary, the combination of atherectomy and percutaneous transluminal angioplasty was observed to have a protective effect against adverse outcomes.

Abnormal glucose metabolism has been recognized as a substantial risk factor for the progression of atherosclerosis [21]. Dysregulated blood glucose can disrupt autonomic function, elevate oxidative stress, inflict damage on the vascular endothelium, and foster the formation of atherosclerotic plaques. Among individuals with diabetes and concurrent ASO, there is a tendency for the involvement of the femoral profunda and infrapatellar arteries in both lower extremities. These affected vessels tend to be small in caliber with extensive branching, predisposing diabetic ASO patients to high rates of restenosis and reocclusion after surgical revascularization [22].

Platelets, which are anuclear cells involved in inflammation, play a significant role in the development and complications of atherosclerosis. Upon activation, these cells release a variety of chemokines, promoting localized inflammation at vascular injury sites. This process results in pathogenic intimal thickening and contributes to vascular obstruction [23]. Additionally, endovascular procedures may trigger thrombus formation. Platelets perceive artificial grafts as foreign entities and undergo activation upon contact with the graft surface. This activation can lead to early postoperative vascular obstruction, particularly when grafts are placed distally to the popliteal artery. Consequently, long-term antiplatelet therapy is often advised for patients receiving artificial vascular grafts [24].

Our study suggests that atherectomy + PTA is a protective factor for poor prognosis in ASO patients undergoing endovascular therapy. Atherectomy entails the physical removal of atherosclerotic plaque from the arterial lumen, thereby reducing the overall plaque burden. This debulking process promotes the creation of a larger and more uniform lumen, facilitating improved blood flow and lowering the risk of restenosis [25]. Furthermore, atherectomy eliminates calcified and fibrotic plaques, enhancing the compliance of the treated vessel segment. Consequently, this improves the efficacy of subsequent PTA by enabling better balloon expansion and more effective dilatation of the vessel. Additionally, the combined use of atherectomy and PTA may induce a more favorable healing response by generating a smoother luminal surface, potentially reducing the likelihood of neointimal hyperplasia, a common cause of restenosis following angioplasty [26].

Regarding the necessity for stent placement, it is crucial to recognize that this decision often reflects the severity and complexity of peripheral vascular disease. Patients requiring stents typically present with advanced disease characterized by extensive and complex lesions prone to elastic recoil or dissection following angioplasty [27]. This necessity underscores a challenging clinical scenario, inherently associated with a higher risk of adverse outcomes.

However, it is crucial to emphasize that the choice of surgical method for ASO patients should always be tailored to the specific conditions of the patient's arteries. The decision-making process must consider various factors, including the extent and location of the arterial lesions, the overall health of the patient, and any comorbid conditions that may influence the outcome of the procedure [28]. Despite these considerations, the results of our study are promising. They indicate that atherectomy + PTA can be highly effective in managing ASO. Future research should focus on further refining patient selection criteria and exploring the long-term benefits of this combined therapeutic approach to maximize patient outcomes.

In conclusion, a majority of patients with ASOs possess multiple risk factors, the cumulative effects of which can accelerate atherosclerotic processes. Our study demonstrated the potential of using the UMLA to identify novel subtypes and construct prediction models in ASO patients receiving endovascular therapy. The current prediction model has the potential to stratify ASO risk, facilitating personalized care through medications, lifestyle adjustments, vigilant monitoring, and timely interventions. Nonetheless, additional validation and enhancement of the model are necessary before its clinical implementation. In summary, the combination of machine learning with precision medicine represents a promising approach to enhance outcomes in patients undergoing endovascular therapy for ASO.

## Conclusions

This study demonstrated that unsupervised machine learning can uncover novel phenotypic subgroups of ASO patients who are receiving endovascular therapy and who are at varying prognostic risk. The prediction model developed could support clinical decision-making and risk counseling for this complex patient population. Further external validation is warranted to assess the generalizability of the findings.

### Supplementary Information

Supplementary Material 1.

## Data Availability

No datasets were generated or analysed during the current study.

## Abbreviations

ABI: Ankle-brachial index
AUC: Area under the curve
ASO: Arteriosclerosis obliterans
BMI: Body mass index
CAD: Coronary artery disease
CVD: Cerebrovascular disease
CKD: Chronic kidney disease
CI: Confidence interval
LDL-C: Low-density lipoprotein cholesterol
MODS: Multiple organ dysfunction syndrome
PTA: Percutaneous transluminal angioplasty
ROC: Receiver operating characteristic
SC: Silhouette coefficient
TASC II: TransAtlantic Inter-Society Consensus II
TC: Total cholesterol
TG: Triglyceride
UMLAs: Unsupervised machine learning algorithms
