# Supplementary material for: Construction of prediction models for novel subtypes in patients with arteriosclerosis obliterans undergoing endovascular therapy: an unsupervised machine learning study
Source: J Cardiothorac Surg. 2024 Jun 25;19:370. doi: 10.1186/s13019-024-02913-6 (PMC11197167; doi:10.1186/s13019-024-02913-6)
Supplement: Supplementary file 1 — Supplementary Material 1. [file 13019_2024_2913_MOESM1_ESM.docx]

Supplementary Table 1: Patient demographics and baseline characteristics

| **Characteristics** | **Training Cohort** | | | **Internal Test Cohort** | | |
| --- | --- | --- | --- | --- | --- | --- |
|  | **Cluster 1**  N = 174 | **Cluster 2**  N = 140 | **p-value** | **Cluster 1**  N = 69 | **Cluster 2**  N = 65 | **p-value** |
| **Gender** |  |  | 0.004 |  |  | 0.218 |
| Female | 80 (46%) | 42 (30%) |  | 26 (38%) | 18 (28%) |  |
| Male | 94 (54%) | 98 (70%) |  | 43 (62%) | 47 (72%) |  |
| **Age** |  |  | 0.003 |  |  | 0.184 |
| Mean ± SD | 58 ± 12 | 62 ± 12 |  | 58 ± 12 | 61 ± 11 |  |
| Median (IQR) | 56 (49, 67) | 63 (52, 70) |  | 57 (49, 68) | 62 (52, 70) |  |
| **BMI** |  |  | 0.035 |  |  | 0.125 |
| Mean ± SD | 22.3 ± 3.6 | 22.9 ± 3.4 |  | 23.0 ± 4.4 | 23.6 ± 3.1 |  |
| Median (IQR) | 21.9 (20.0, 24.4) | 22.7 (20.4, 25.4) |  | 22.3 (19.8, 25.7) | 23.5 (21.9, 25.6) |  |
| **Smoking** |  |  | 0.002 |  |  | 0.153 |
| No | 76 (44%) | 38 (27%) |  | 26 (38%) | 17 (26%) |  |
| Yes | 98 (56%) | 102 (73%) |  | 43 (62%) | 48 (74%) |  |
| **Hypertension** |  |  | <0.001 |  |  | 0.317 |
| No | 136 (78%) | 72 (51%) |  | 42 (61%) | 34 (52%) |  |
| Yes | 38 (22%) | 68 (49%) |  | 27 (39%) | 31 (48%) |  |
| **Diabetes** |  |  | <0.001 |  |  | 0.322 |
| No | 147 (84%) | 92 (66%) |  | 49 (71%) | 51 (78%) |  |
| Yes | 27 (16%) | 48 (34%) |  | 20 (29%) | 14 (22%) |  |
| **CAD** |  |  | 0.616 |  |  | 0.745 |
| No | 163 (94%) | 133 (95%) |  | 63 (91%) | 61 (94%) |  |
| Yes | 11 (6%) | 7 (5%) |  | 6 (9%) | 4 (6%) |  |
| **CVD** |  |  | 0.196 |  |  | >0.999 |
| No | 162 (93%) | 135 (96%) |  | 64 (93%) | 60 (92%) |  |
| Yes | 12 (7%) | 5 (4%) |  | 5 (7%) | 5 (8%) |  |
| **CKD** |  |  | 0.409 |  |  | 0.494 |
| No | 157 (90%) | 130 (93%) |  | 63 (91%) | 57 (88%) |  |
| Yes | 17 (10%) | 10 (7%) |  | 6 (9%) | 8 (12%) |  |
| **TC** |  |  | 0.026 |  |  | 0.025 |
| Mean ± SD | 7.5 ± 3.6 | 8.6 ± 4.2 |  | 7.4 ± 3.6 | 9.0 ± 4.6 |  |
| Median (IQR) | 6.7 (5.1, 9.1) | 7.5 (5.7, 10.3) |  | 6.6 (5.3, 8.4) | 7.6 (6.0, 10.3) |  |
| **Platelet** |  |  | 0.084 |  |  | 0.039 |
| Mean ± SD | 211 ± 77 | 245 ± 127 |  | 207 ± 85 | 252 ± 117 |  |
| Median (IQR) | 209 (154, 255) | 224 (161, 279) |  | 208 (151, 264) | 230 (176, 297) |  |
| **LDL-C** |  |  | 0.060 |  |  | 0.058 |
| Mean ± SD | 5.56 ± 2.61 | 6.61 ± 3.84 |  | 4.99 ± 2.77 | 6.21 ± 3.63 |  |
| Median (IQR) | 5.02 (3.98, 7.27) | 5.90 (3.78, 8.68) |  | 4.13 (2.98, 6.32) | 5.73 (3.31, 7.57) |  |
| **TG** |  |  | 0.741 |  |  | 0.786 |
| Mean ± SD | 1.54 ± 0.64 | 1.49 ± 0.58 |  | 1.55 ± 0.69 | 1.51 ± 0.69 |  |
| Median (IQR) | 1.56 (1.02, 1.89) | 1.50 (1.12, 1.84) |  | 1.56 (1.14, 1.85) | 1.52 (0.97, 1.90) |  |
| **ABI** |  |  | <0.001 |  |  | <0.001 |
| Mean ± SD | 0.65 ± 0.13 | 0.40 ± 0.14 |  | 0.64 ± 0.13 | 0.37 ± 0.16 |  |
| Median (IQR) | 0.67 (0.56, 0.75) | 0.40 (0.31, 0.46) |  | 0.66 (0.54, 0.72) | 0.35 (0.25, 0.44) |  |
| **Rutherford** |  |  | <0.001 |  |  | <0.001 |
| Mean ± SD | 2.32 ± 0.77 | 4.53 ± 1.27 |  | 2.42 ± 0.74 | 4.69 ± 1.35 |  |
| Median (IQR) | 2.00 (2.00, 2.00) | 5.00 (4.00, 5.00) |  | 2.00 (2.00, 3.00) | 5.00 (4.00, 6.00) |  |
| **TASC II** |  |  | <0.001 |  |  | <0.001 |
| A | 89 (51%) | 2 (1%) |  | 32 (46%) | 2 (3%) |  |
| B | 76 (44%) | 10 (7%) |  | 31 (45%) | 4 (6%) |  |
| C | 9 (5%) | 86 (61%) |  | 6 (9%) | 31 (48%) |  |
| D | 0 (0%) | 42 (30%) |  | 0 (0%) | 28 (43%) |  |
| **Operative method** |  |  | <0.001 |  |  | <0.001 |
| PTA | 27 (16%) | 73 (52%) |  | 11 (16%) | 36 (55%) |  |
| Atherectomy+PTA | 88 (51%) | 33 (24%) |  | 36 (52%) | 8 (12%) |  |
| Atherectomy+PTA+Stent | 59 (34%) | 34 (24%) |  | 22 (32%) | 21 (32%) |  |
| **Operation time** |  |  | 0.004 |  |  | 0.008 |
| ＜2h | 57 (33%) | 37 (26%) |  | 19 (28%) | 15 (23%) |  |
| 2~4h | 88 (51%) | 57 (41%) |  | 36 (52%) | 21 (32%) |  |
| >4h | 29 (17%) | 46 (33%) |  | 14 (20%) | 29 (45%) |  |

ABI: Ankle brachial index; BMI: Body mass index; CAD: Coronary artery disease; CVD:Cerebrovascular disease; CKD: Chronic kidney disease; LDL-C: Low-density lipoprotein cholesterol; PTA: Percutaneous transluminal angioplasty; TASC II: TransAtlantic Inter-Society Consensus II; TC: Total cholesterol; TG: triglyceride.
